# Integrated pipeline for inferring the evolutionary history of a gene family embedded in the species tree: a case study on the STIMATE gene family

**DOI:** 10.1186/s12859-017-1850-2

**Published:** 2017-10-03

**Authors:** Jia Song, Sisi Zheng, Nhung Nguyen, Youjun Wang, Yubin Zhou, Kui Lin

**Affiliations:** 1 MOE Key Laboratory for Biodiversity Science and Ecological Engineering, College of Life Sciences, Beijing Normal University, Beijing, 100875 China; 2 Beijing Key Laboratory of Gene Resources and Molecular Development College of Life Sciences, Beijing Normal University, Beijing, 100875 China; 3 Center for Translational Cancer Research, Institute of Biosciences and Technology, Department of Medical Physiology, College of Medicine, Texas A&M University, Houston, TX 77030 USA

**Keywords:** Evolutionary history, Gene family, Phylogenetic tree, STIMATE, Chordate

## Abstract

**Background:**

Because phylogenetic inference is an important basis for answering many evolutionary problems, a large number of algorithms have been developed. Some of these algorithms have been improved by integrating gene evolution models with the expectation of accommodating the hierarchy of evolutionary processes. To the best of our knowledge, however, there still is no single unifying model or algorithm that can take all evolutionary processes into account through a stepwise or simultaneous method.

**Results:**

On the basis of three existing phylogenetic inference algorithms, we built an integrated pipeline for inferring the evolutionary history of a given gene family; this pipeline can model gene sequence evolution, gene duplication-loss, gene transfer and multispecies coalescent processes. As a case study, we applied this pipeline to the STIMATE (TMEM110) gene family, which has recently been reported to play an important role in store-operated Ca^2+^ entry (SOCE) mediated by ORAI and STIM proteins. We inferred their phylogenetic trees in 69 sequenced chordate genomes.

**Conclusions:**

By integrating three tree reconstruction algorithms with diverse evolutionary models, a pipeline for inferring the evolutionary history of a gene family was developed, and its application was demonstrated.

**Electronic supplementary material:**

The online version of this article (10.1186/s12859-017-1850-2) contains supplementary material, which is available to authorized users.

## Background

Within a group of related species of interest, an accurate phylogenetic tree of a given gene family underpins either a valid inference of its evolutionary history or a correct understanding of its biological function [1–4]. To date, many if not most gene family trees have been reconstructed only by modelling the respective sequence evolution [5–8]. However, in spite of this method’s great success in molecular phylogenetics, many studies [9, 10] have suggested that this category of ‘sequence only’ methods is confounded because most gene sequences lack sufficient information to confidently support one gene tree over another. Theoretically, coestimation of the gene family tree and the species tree is an ideal approach, owing to the rationale is that all gene families are evolving embedded in the species tree, even though they may differ from the species tree because of the effect of a hierarchy of evolutionary processes [10–12]. Currently, this category of phylogenetic inferences is often intractable because of limited computational capacity [13, 14].

Thus, a third category of computational methods, collectively known as “species tree aware”, has been proposed and developed in the past few years. Several methods [9, 15–18] have been developed to date to implement this idea successfully to infer the evolutionary history of a gene family evolved and embedded in a given species tree. For example, ALE (amalgamated likelihood estimation) is an algorithm implementing a birth-death process to model gene duplication, loss and transfer to infer a gene family tree [17]. Furthermore, *BEAST (Bayesian evolutionary analysis by sampling trees) can infer phylogenetic gene trees embedded in the species tree by modelling a multispecies coalescent process [18]. As an alternative, several methods have been developed to use species tree information to correct the gene tree [19–21]. These methods are usually based on a reconciliation framework and attempt to minimize a species tree aware cost function based on the inferred evolutionary events. Obviously, these approaches are considerably simpler than model-based species tree aware approaches.

Currently, to the best of our knowledge, there is no single algorithm or existing tool that can infer gene family trees while taking into account all four evolutionary events, namely, duplication, loss, transfer and incomplete lineage sorting (ILS) [22]. In addition, from the viewpoint of evolutionary genomics, biologists are more interested in accurately analysing a set of functionally related gene families over a single family. To this end, we set out to develop an integrative analysis pipeline mainly based on the ALE, BEAST [23] and *BEAST tools to accelerate a more accurate inference of evolutionary history for a gene family. As a case study, we explored the evolutionary histories of the STIMATE gene family and the families of its possible co-players stromal interaction molecule (STIM) and calcium release-activated calcium modulator (ORAI) [24–27]. STIMATE has been shown to interact with STIM proteins, which are mediators of store-operated Ca^2+^ entry (SOCE), and to play crucial regulatory roles in mediating calcium signalling occurring at ER-PM junctions [26, 27]. Our results demonstrated that this pipeline was highly efficient in reconstructing the evolutionary history of a given gene family, as exemplified by the STIMATE genes.

## Results

### Integrated pipeline for inferring the evolutionary history of a gene family embedded in the species tree

**Fig. 1:**
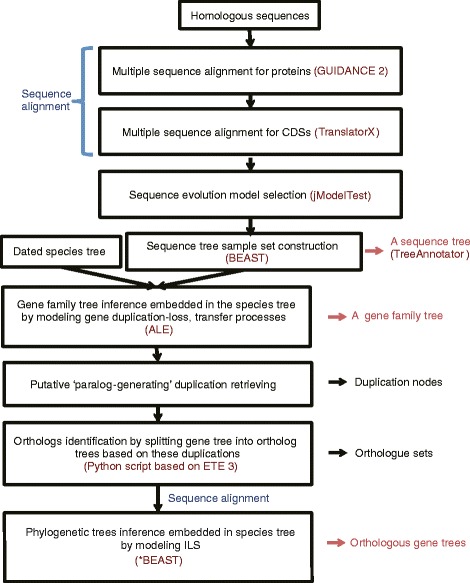
Flowchart illustrating our integrated pipeline. By integrating two alignment tools and three phylogenetic inference methods, we aimed to infer the gene family tree and the orthologous gene tree(s) with high accuracy

In Fig. 1, by integrating two sequence alignment tools (GUIDANCE 2 [28] and TranslatorX [29]) and three gene tree inference algorithms (BEAST and *BEAST, implemented in BEAST 2, and ALE [14]), we designed our pipeline to explore the evolutionary histories of gene families. First, by using the BEAST algorithm (the basic module of BEAST 2), we estimated a rooted, time-measured gene family tree sample set from the respective posterior distribution using various substitution, site and molecular clock models. Second, on the basis of this sample set and the dated species tree, a gene family tree was inferred by using the ALE approach, which enables the combination of the estimation of sequence likelihood with probabilistic reconciliation methods. Next, we retrieved this gene family tree to find the putative ‘paralog-generating’ nodes with left and right sub-trees containing two or more common species. On the basis of these nodes, the gene family tree was split into ortholog trees with our python scripts based on ETE 3 [30] to obtain orthologue sets. Furthermore, phylogenetic trees of these orthologue sets were reconstructed in *BEAST (another modular of BEAST 2) on the basis of the multispecies coalescent model. By comparing the results from all these steps, we obtained an overall view of the evolution of the gene family.

As a case study, we used the STIMATE gene family to test our pipeline. This gene family consists of 81 members from 69 species. After sequence alignment and trimming processes, which are included in our pipeline, we obtained a CDS MSA (multiple sequence alignment) with 975 bp. Using one CPU core, this analysis required approximately 2 h for BEAST to generate a gene tree sample set with 20,000 trees, approximately 0.5 h for ALE to generate the gene family tree and approximately 80 h for *BEAST to generate a tree posterior distribution sample set with 500,000 trees for each ortholog. The running time of BEAST and *BEAST can be decreased significantly by using multiple CPU cores to run multiple chains (e.g., ~ 3 h for *BEAST with 30 CPU cores on our computing system). Therefore, our pipeline can use the CDS sequence from species with larger evolutionary scales to infer gene family trees embedded in the species tree within an acceptable running time.

### Gene family trees of STIMATE

**Fig. 2:**
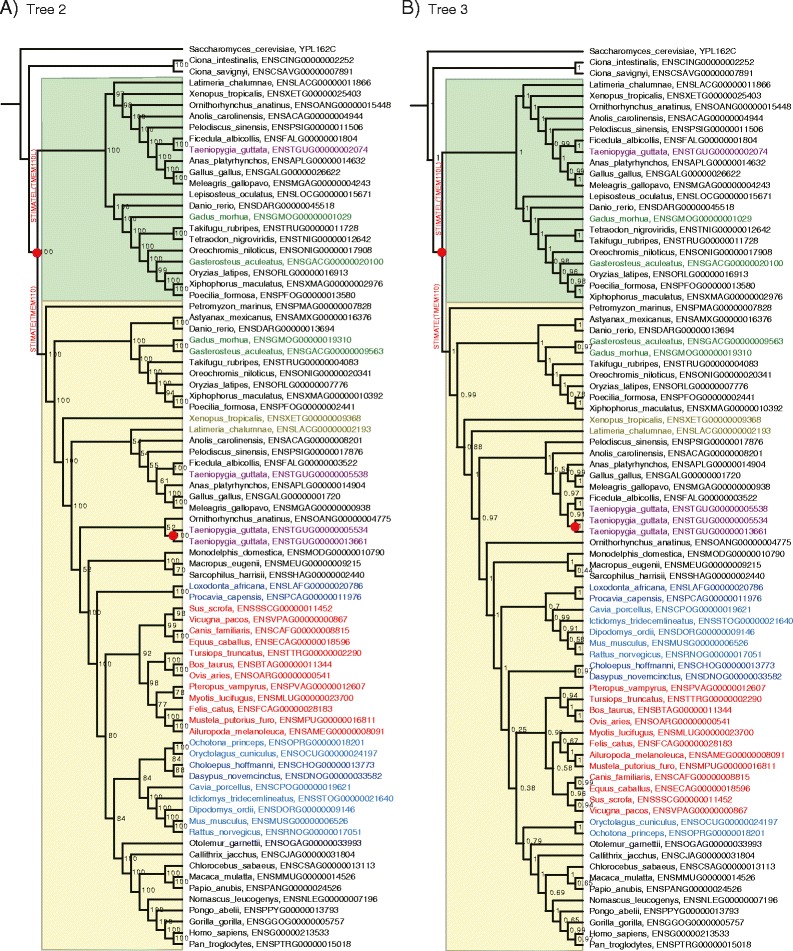
STIMATE gene family trees generated by our pipeline. The nodes annotated with red dots are the gene duplication nodes. The names of leaves affected by phylogenetic incongruence between the gene trees and the species tree are labelled in colours other than black. **a** Tree 2. The STIMATE gene family tree resulting from ALE in our pipeline. The node labels are the bootstrap values. **b** Tree 3. The STIMATE gene family tree resulting from *BEAST in our pipeline. The node labels are the posterior probabilities

With the gene family tree sample set derived by BEAST, a gene family tree with maximum clade credibility (Additional file 1a: Tree 1) was obtained with the TreeAnnotator programme, which summarized the tree sample set representing the gene evolutionary history reflected solely by sequence data. After analysis using the DTL model in ALE with the species phylogeny and the tree sample set, we obtained another gene family tree (Tree 2, Fig. 2a). Splitting at the unique ‘paralog-generating’ node located before the divergence of lampreys on Tree 2, two orthologous gene sets were established, and two phylogenetic trees were separately reconstructed in *BEAST. Next, these two orthologous gene trees were combined as Tree 3 (Fig. 2b). In addition, we also downloaded the corresponding STIMATE gene family tree from Ensembl 83 (Additional file 1b: Tree 4).

We compared these four gene family trees according to their maximum log likelihoods based on the CDS MSAs and their average normalized RF (Robinson-Foulds) distances [31] from the species tree (Table 1). Tree 3, the final gene family tree of our pipeline, appeared to have the highest maximum likelihood either on the basis of the MSA generated by our pipeline or the MSA downloaded from Ensembl. Unexpectedly, this tree’s likelihood was even greater than that of Tree 1. With respect to RF distance, Tree 2 bore the smallest value (0.12) from the species tree among these four trees. Tree 3 (0.14) was comparable to Tree 2, whereas Tree 4 and Tree 1 had larger RF values (Column 2 in Table 1). These values showed that the gene family trees generated by our pipeline (Tree 2 and Tree 3) might reflect a more accurate evolutionary history than either Tree 1 (sequence only) or Tree 4 (Ensembl). In addition, we also reconstructed the gene family trees of STIM and ORAI, which were considered putative co-players with STIMATE (Additional files 2 and 3).

**Table 1:** Gene tree maximum log likelihoods based on MSAs and nRF distance from the species tree

Tree	Description	nRF^a^	LogL1^b^	LogL2^c^
Tree 1	Sequence only	0.31	−28,470	−31,425
Tree 2	ALE following BEAST	0.12	−28,479	−31,456
Tree 3	*BEAST following ALE and BEAST	0.14	−28,462	−31,423
Tree 4	Ensembl	0.21	−28,585	−31,536

^a^ETE 3 was used to estimate the average nRF (normalized RF) distance between the gene family tree and the species tree

^b^The maximum log likelihoods of gene trees were estimated on the basis of the MSA generated by our pipeline

^c^The maximum log likelihoods of gene trees were estimated on the basis of the MSA downloaded from Ensembl 83

*BEAST or StarBeast

### Evolutionary history of the STIMATE genes

**Fig. 3:**
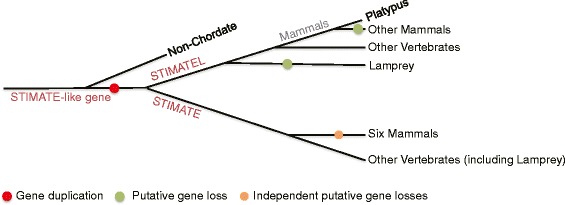
Main gene duplications and losses derived from the STIMATE gene family tree

On the basis of the inferred STIMATE gene family trees, the primary STIMATE family expansion and contraction histories are summarized in Fig. 3a putative duplication occurred at the beginning of chordate genome evolution before the divergence of lampreys and gnathostomes, and might have resulted in the origin of STIMATE and its paralog named STIMATEL (or TMEM110L) herein. Likewise, some putative loss events contributed to the complete evolutionary history of the STIMATE family. For example, STIMATEL was lost in the genomes of mammals (except for the platypus, a semiaquatic egg-laying mammal) and lampreys after this duplication event. Inexplicably, the STIMATE genes were not found in two non-chordate model species genomes (*Caenorhabditis elegans* and *Drosophila melanogaster*) and six mammalian genomes (*Tarsius syrichta*, *Microcebus murinus, Tupaia belangeri, Erinaceus europaeus, Sorex araneus,* and *Echinops telfairi)*. Presumably, these eight independent absences might also have been caused by gene loss.

In addition, there were several incongruences among the STIMATE gene family trees (Tree 2 and Tree 3, Fig. 2) inferred on the basis of different models in our pipeline and the species tree (Additional file 4). The clades showing incongruence between the gene family trees inferred by our pipeline and the species tree are labelled on the trees. Furthermore, the relative clades are labelled in Additional file 1: Tree 1. A previous study [32] has indicated that there are various biological factors (lineage sorting, horizontal gene transfer, gene duplication and loss, hybridization, recombination, natural selection and other more complex mechanisms) that can cause incongruence. To distinguish these causes, we compared the incongruences labelled on these three trees (Tree 1, Tree 2 and Tree 3) and aimed to explore the evolutionary history of the STIMATE gene family in the chordate genomes (discussed in Additional file 5).

## Discussion

### Advantages of our phylogenetic inference pipeline

Our pipeline may provide more opportunities to obtain accurate gene family trees that contain more information on the evolutionary histories of gene families.

First, we generated a CDS MSA guided by a protein MSA. The protein MSA was generated by GUIDANCE2, which considers that alignments vary substantially when given alternative tree topologies to guide the progressive alignment and calculates guidance scores. We tested several cutoff values during the guidance score-based MSA column filtering process and chose 0.5 as a cutoff value instead of the default value of 0.93 according to the evolutionary distance among the 69 species. All of these manipulations strengthen the reliability of the alignment and save computation time. Meanwhile, in our pipeline, a choice can be made to filter or not filter before any phylogenetic inferences are drawn. More details of the filtering cutoff selection procedure (including comparisons with unfiltered sequences) are listed in Additional file 6.

Second, our inference procedure takes into account three algorithms for modelling different evolutionary processes/events at different levels. The gene family evolution model exODT [33] integrated into ALE [17] considers various gene family evolution events (speciation and extinction at the species level, gene duplication, loss and transfer at the genome level). Although horizontal gene transfer is expected to be very rare or absent in animals [32], this model is a better choice to avoid the overestimation of gene duplication and loss, and it helps to retain more real incongruence attributable to evolutionary events between the gene family tree and species tree. Next, by taking a tree sample from a BEAST analysis and a given species tree as input, ALE allows for reconstruction of a gene family tree that maximizes the product of the probability of the alignment given the gene family tree and the probability of the gene family tree given the species tree. Further, the cooperation of BEAST and ALE allowed us to use more sequence evolution models than algorithms such as SPIMAP [9] or PRIME-GSR [34], which directly infer gene trees by using an MSA under a given species tree. The latter generally has more strict data requirements in real applications. For example, SPIMAP requires training data, which are difficult to obtain in our test. Further, on the basis of the ALE results, *BEAST [18] infers the gene tree for the orthologous gene sequences by using a multispecies coalescent model, which can model evolutionary processes at the sequence, population and species levels. This gene tree should aid in identifying the clades affected by ILS. Therefore, the inference procedure in our pipeline is expected to accurately identify putative evolutionary events from the species, population, genome and sequence site levels.

The BEAST and *BEAST steps in our pipeline can be substituted with other algorithms, but they are recommended because of their convenience in pipeline construction. Because BEAST and *BEAST are two modules in BEAST 2, installing BEAST 2 and ALE is sufficient for our platform. BEAST 2 is a well-established cross-platform programme that is easy to install. In addition, BEAST is very efficient in generating large tree samples. With our preliminary comparison using the STIMATE dataset, BEAST was approximately ten times faster than PhyloBayes [35]. Users can also substitute BEAST and *BEAST with other tools. For example, PhyloBayes may contain relatively complicated evolutionary models (such as CAT), which have not yet been included in BEAST. This substitution is simple in our pipeline. In this study, we compared the potential performance of some tools used in our pipeline with those of other similar algorithms. The detailed comparisons among these results are presented in Additional file 7.

### Limitations and future development of our pipeline

In this study, our pipeline was designed to consider gene duplication, loss, transfer and ILS in a stepwise manner, which may be inconsistent with real evolutionary scenarios. Thus, future development for our pipeline should focus on methods that can model such different factors simultaneously. Next, to greatly decrease the computational complexity, the topology of the species tree should be fixed and assigned beforehand, and could be, for example, downloaded from a reliable database, such as Ensembl [36]. Certainly, this configuration may limit our pipeline’s ability to infer a larger scale gene family tree if there is no extant or well-known species tree. These shortcomings will be alleviated by incorporating efficient species tree inference tools into our pipeline in the near future. In addition, we will integrate gene expression and synteny block information into our pipeline in the future, because such data may help us to characterize the causes of the incongruence between the inferred phylogenetic trees.

## Conclusions

Primarily using three tree reconstruction algorithms that consider different evolutionary events, we developed an integrated pipeline to infer an accurate evolutionary history of a given gene family. Next, we used STIMATE as a case study to demonstrate a complete application of our pipeline on the accurate inference of the evolutionary history of the STIMATE gene family in sequenced chordate genomes. We believe that our pipeline should facilitate further studies aiming to explore accurate gene family evolutionary history, particularly in the genomes of model species.

## Methods

We developed a phylogenetic inference procedure to infer gene trees embedded in a given species tree. Our analysis pipeline is shown in Fig. 1. Here, we used the STIMATE gene family as a case study.

### Species tree dating

We downloaded the species tree including 69 species from Ensembl (http://asia.Ensembl.org/info/about/speciestree.html) [36]. This tree describes the evolutionary relationship of 43 mammals, 5 birds, 2 reptiles, 1 amphibian, 12 fish, 3 other chordates and 3 non-chordate model species. To date this species tree, we downloaded all CDS and protein sequences of these 69 species from Ensembl. After clustering these genes into different families using OrthoFinder [37], we found 26 gene families with a single copy in most species (> = 68 species). These 26 gene families were then used to date the species tree by using *BEAST (parameters: fixed topology of species tree, a gamma-distributed model of rate variation with four discrete categories and an HKY substitution model with a strict clock) after aligning with MAFFT [38] and trimming with trimAL (−gt 0.5 –st 0.001 -cons 50) [39].

### Sequence alignment

According to the human STIMATE gene (ENSG00000213533), a list of protein IDs containing all STIMATE protein family members in the 69 species from Ensembl release 83 was retrieved. The respective CDS and protein sequences were then downloaded by using the Ensembl Perl API.

A MSA of the downloaded protein sequences was generated by using the MAFFT [38] algorithm implemented in GUIDANCE2 [28] with 100 iterations (−-MSA_Param “\--maxiterate 100” --bootstraps 100). A CDS MSA was subsequently generated under the guidance of this protein MSA using TranslatorX [29]. We removed the columns whose respective guidance scores were below 0.5 after considering the conservative property of our data (see Additional file 6).

### Phylogenetic tree inference

On the basis of the well-aligned CDS sequences of the STIMATE family, BEAST v2.3.0 [14] was first used to generate a sample of gene family trees (20,000,000 generations, sampling every 1000 generations). Here, the substitution model was selected by jModelTest v2.1.7 [40, 41]. The inferred tree sample set and our dated species tree were then used as inputs to ALE [17] to obtain a gene family tree (bootstraps: 1000).

In general, on the gene family tree, most nodes that exist in only one common species between their left and right sub-trees are species-specific duplication nodes. To both control the number of orthologue sets and to avoid including too many paralogs in any orthologue set, the Species Overlap (SO) algorithm [42] was used to retrieve the ALE gene family tree and define nodes as ‘paralog-generating’ nodes, whose left and right sub-trees contained two or more common species. We found only one such ‘paralog-generating’ node on the STIMATE gene family tree inferred with ALE. By splitting by this node we obtained two orthologue sets with 61 and 23 members, respectively. As an alternative, we also implemented the reconciliation algorithm in ETE 3 [30, 43] in our pipeline for users who wish to find all putative duplications.

After generating the CDS alignments with GUIDANCE2 and TranslatorX, we used *BEAST [18] to reconstruct a STIMATE ortholog tree and a STIMATEL ortholog tree embedded in our species tree with a fixed topology (parameters: ~500,000,000 generations, sampling every 1000 generations, General Time Reversible model coupled with a gamma-distributed model of rate variation with four discrete categories, Log Normal Relaxed Clock [44]).

The STIM/ORAI CDS MSA, gene family tree and ortholog trees were inferred in the same way.

### Trees comparison

We compared four STIMATE gene family trees according to their log likelihoods based on the CDS MSAs and their average normalized RF (Robinson-Foulds) distances [31] from the species tree. The maximum log likelihoods of these trees based on the CDS MSAs were directly estimated by using IQ-TREE [45]. The average normalized RF distances between the gene family trees and the species tree were estimated with an approach similar to TreeKO [46]. We first split the gene family tree into two ortholog trees (the STIMATE tree and the STIMATEL tree). For each of these two ortholog trees, we used an SO algorithm [30, 42] (the species overlap score threshold was set to 0.0) to find putative duplications. On the basis of these putative duplications, the orthologous gene tree was split into species trees. The normalized RF distances between these trees and the species tree was estimated by using ETE 3 [30]. For each ortholog tree, the average normalized RF distance was then estimated, and the average normalized RF distance between the STIMATE gene family tree and the species tree was obtained.

## Additional files

Additional file 1: Gene trees of STIMATE. A) STIMATE gene family tree (Tree 1) from TreeAnnotator. The node labels are the posterior probabilities. B) STIMATE gene family tree downloaded from Ensembl. (PDF 115 kb)

Additional file 2: STIM gene family and orthologous gene trees. (PDF 403 kb)

Additional file 3: ORAI gene family and orthologous gene trees. (PDF 348 kb)

Additional file 4: Dated species tree of 69 species. (PDF 61 kb)

Additional file 5: Evolutionary history of the STIMATE gene family. (PDF 4081 kb)

Additional file 6: Alignment filtering cutoff choice and comparison. (PDF 2385 kb)

Additional file 7: Comparison with Phylobayes and TERA. (PDF 51 kb)

## Abbreviations

ALE: Amalgamated likelihood estimation
BEAST: Bayesian evolutionary analysis by sampling trees
CDS: Coding DNA sequence
GTR: Generalized time reversible
HKY: Hasegawa, Kishino and Yano (a substitution model)
ILS: Incomplete lineage sorting
MAFFT: Multiple alignment using fast fourier transform
MCMC: Markov chain monte carlo
MSA: Multiple sequence alignment
MUSTN: Musculoskeletal, embryonic nuclear protein
ORAI: Calcium release-activated calcium modulator
SOCE: Store-operated Ca^2+^ entry
STIM: Stromal interaction molecule
STIMATE (TMEM110): Transmembrane protein 110
STIMATEL (TMEM110L): Transmembrane protein 110, Like
