# Supplementary material for: Integrated pipeline for inferring the evolutionary history of a gene family embedded in the species tree: a case study on the STIMATE gene family
Source: BMC Bioinformatics. 2017 Oct 3;18:439. doi: 10.1186/s12859-017-1850-2 (PMC5627428; doi:10.1186/s12859-017-1850-2)

Gene trees of STIMATE

The nodes annotated with red dots are the gene duplication nodes. The names of leaves affected by the phylogenetic incongruence between Tree 2, Tree 3 and the species tree are labeled in colors other than black on Tree 1.

A) Tree 1

The sequence tree of STIMATE gene family inferred by BEAST.

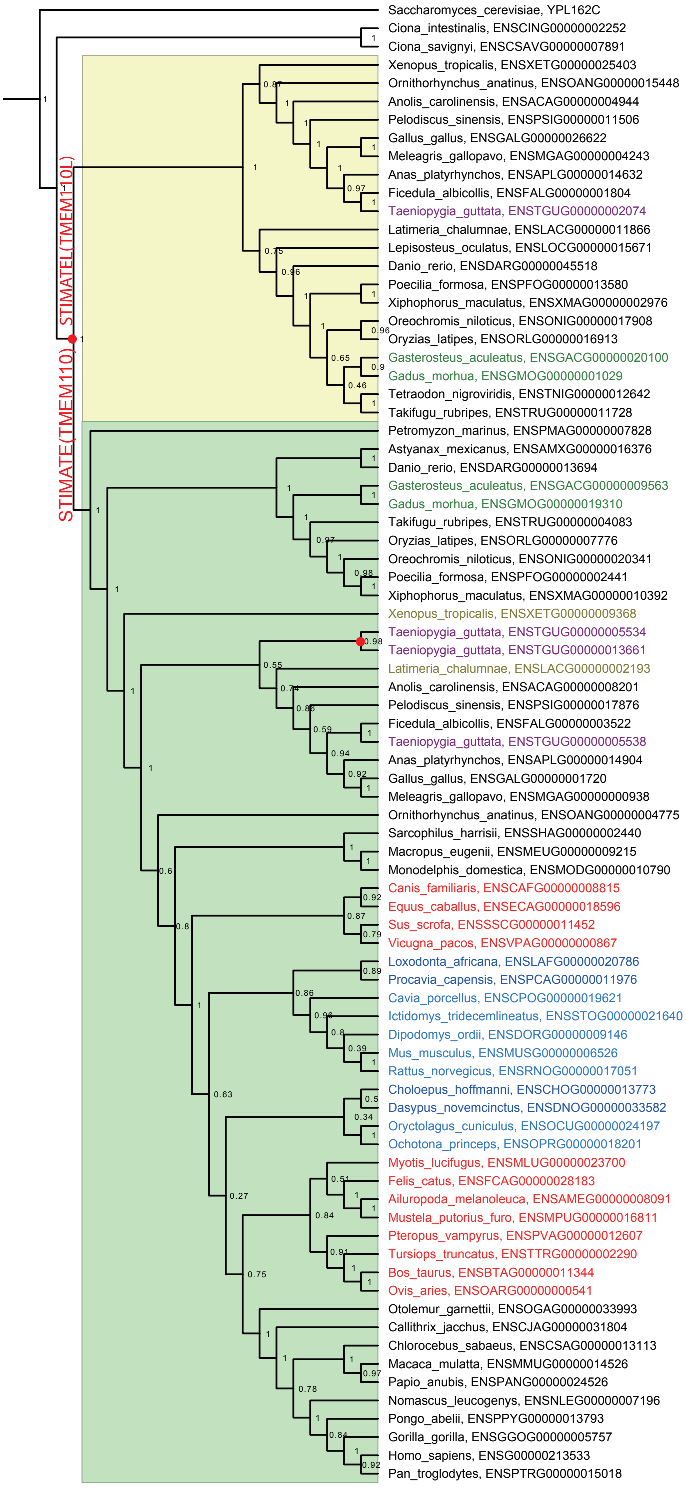

B) Tree 4

The STIMATE gene family tree downloaded from Ensembl.

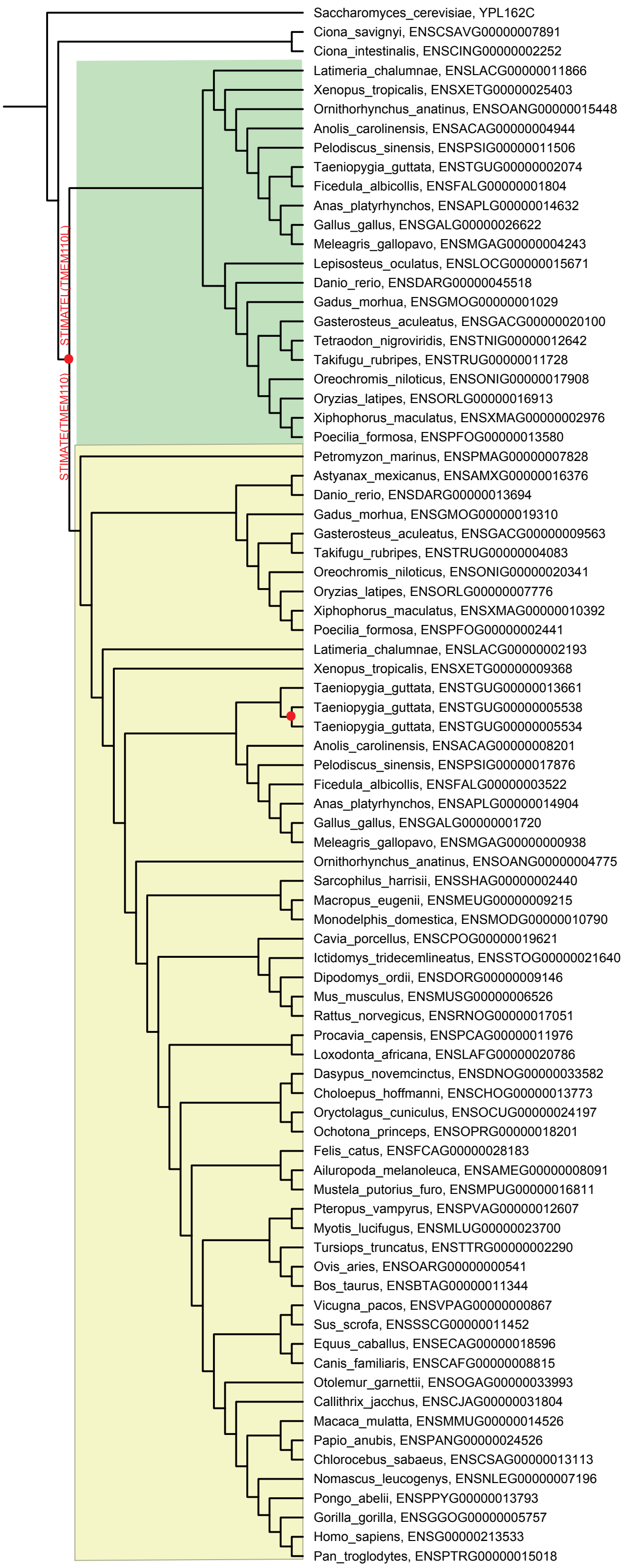

Supplement: Supplementary file 1 — Gene trees of STIMATE. A) STIMATE gene family tree (Tree 1) from TreeAnnotator. The node labels are the posterior probabilities. B) STIMATE gene family tree downloaded from Ensembl. (PDF 115 kb) [file 12859_2017_1850_MOESM1_ESM.pdf]
