# Supplementary material for: Integrated pipeline for inferring the evolutionary history of a gene family embedded in the species tree: a case study on the STIMATE gene family
Source: BMC Bioinformatics. 2017 Oct 3;18:439. doi: 10.1186/s12859-017-1850-2 (PMC5627428; doi:10.1186/s12859-017-1850-2)

Gene family tree and orthologous gene trees of STIM

The node labels are the bootstrap values in A and posterior probabilities in B, C, D and E. Gene duplication nodes are annotated with red dots. The leaves highlighted in clover are two species that show similar incongruence between the gene trees and species tree as STIMATE-like gene.

A) The gene family tree of STIM (resulted from ALE following BEAST)

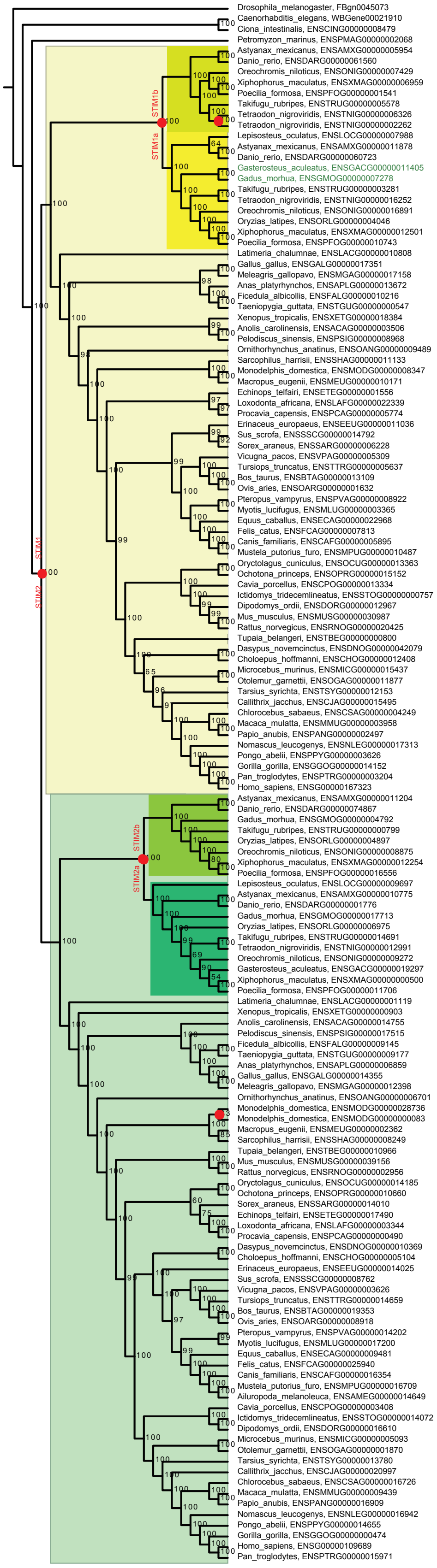

B) Tree of orthologue set 1/ STIM 1b (resulted from *BEAST)

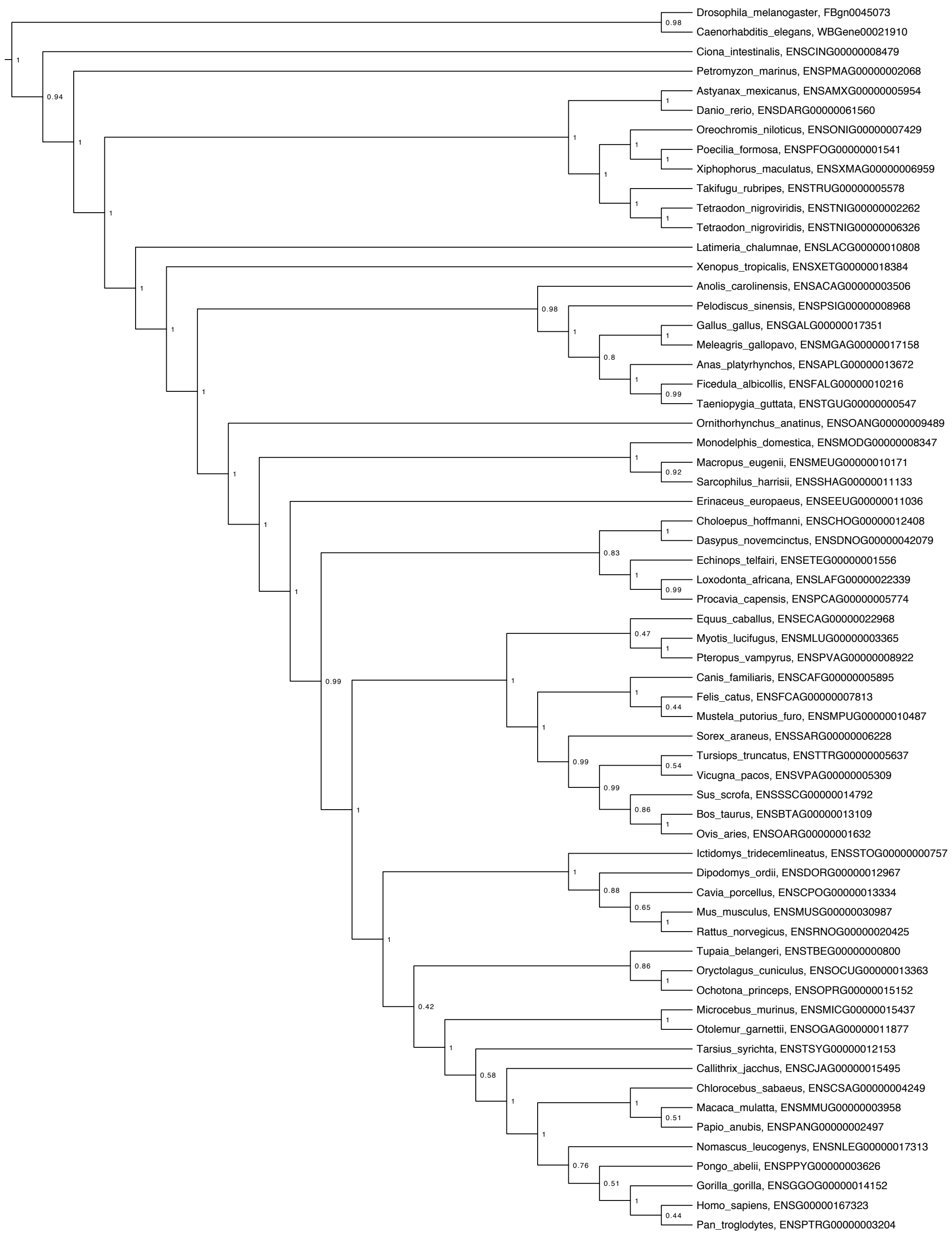

C) Tree of orthologue set 2/ STIM 1a (resulted from *BEAST)

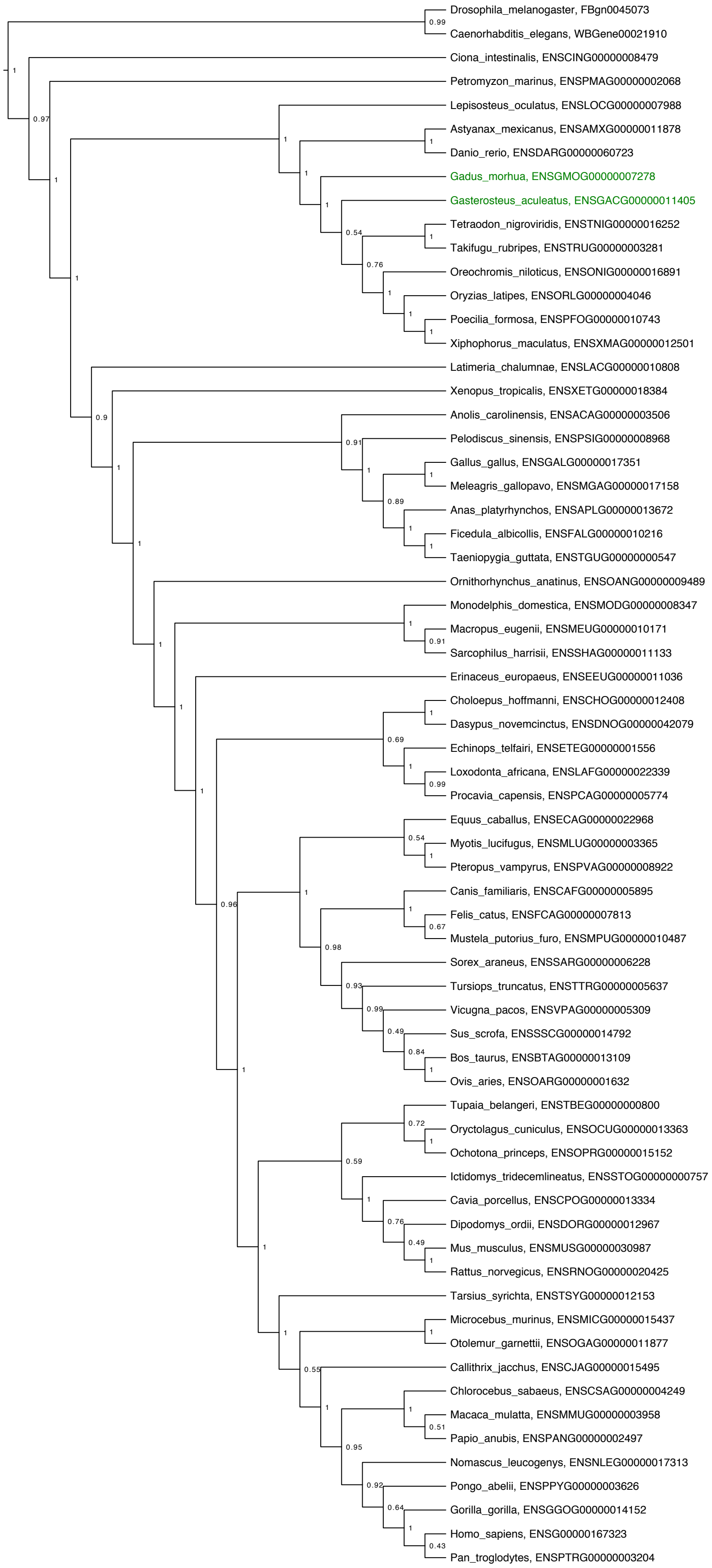

D) Tree of orthologue set 3/ STIM 2b (resulted from *BEAST)

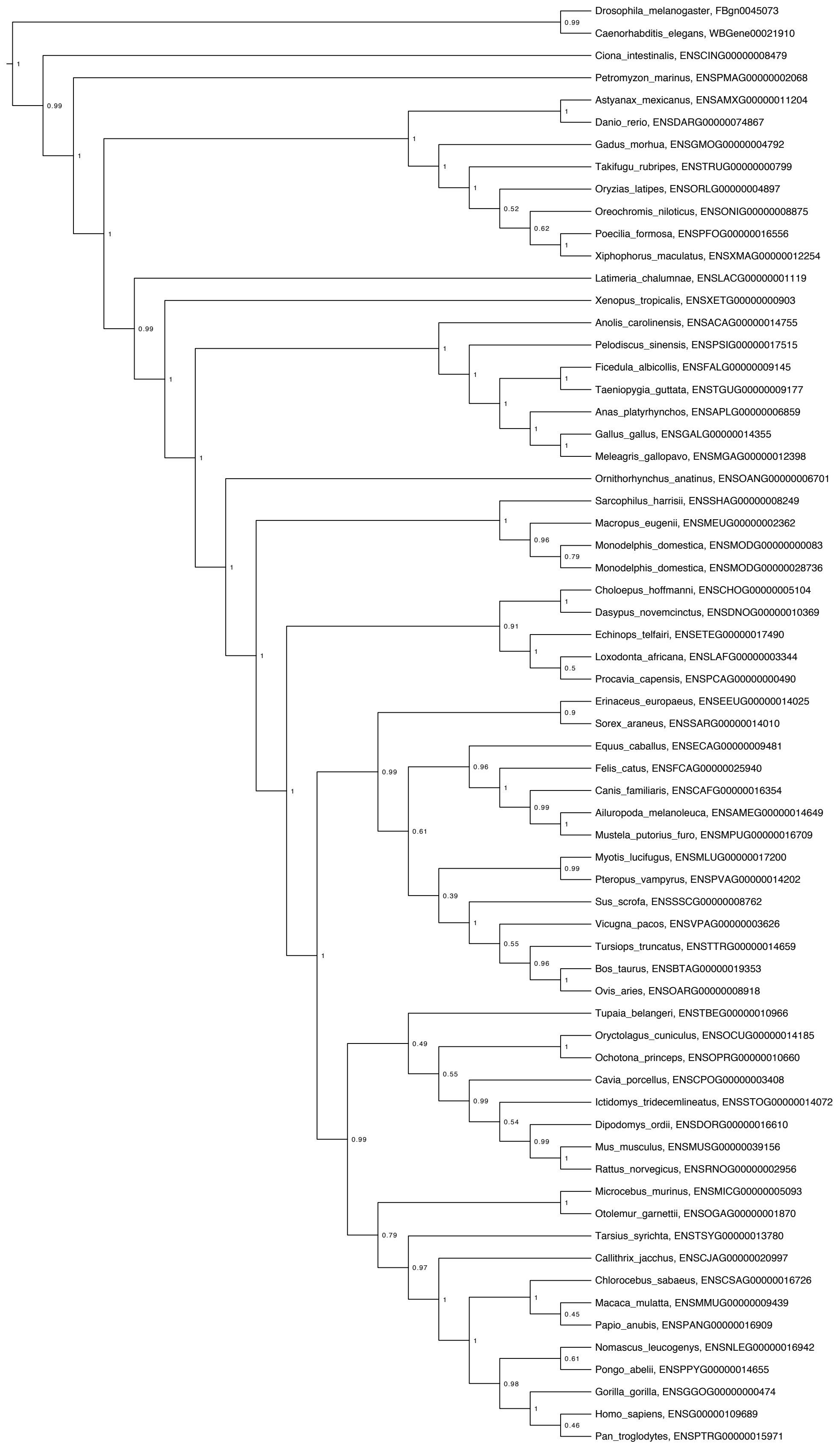

E) Tree of orthologue set 4/ STIM 2a (resulted from *BEAST)

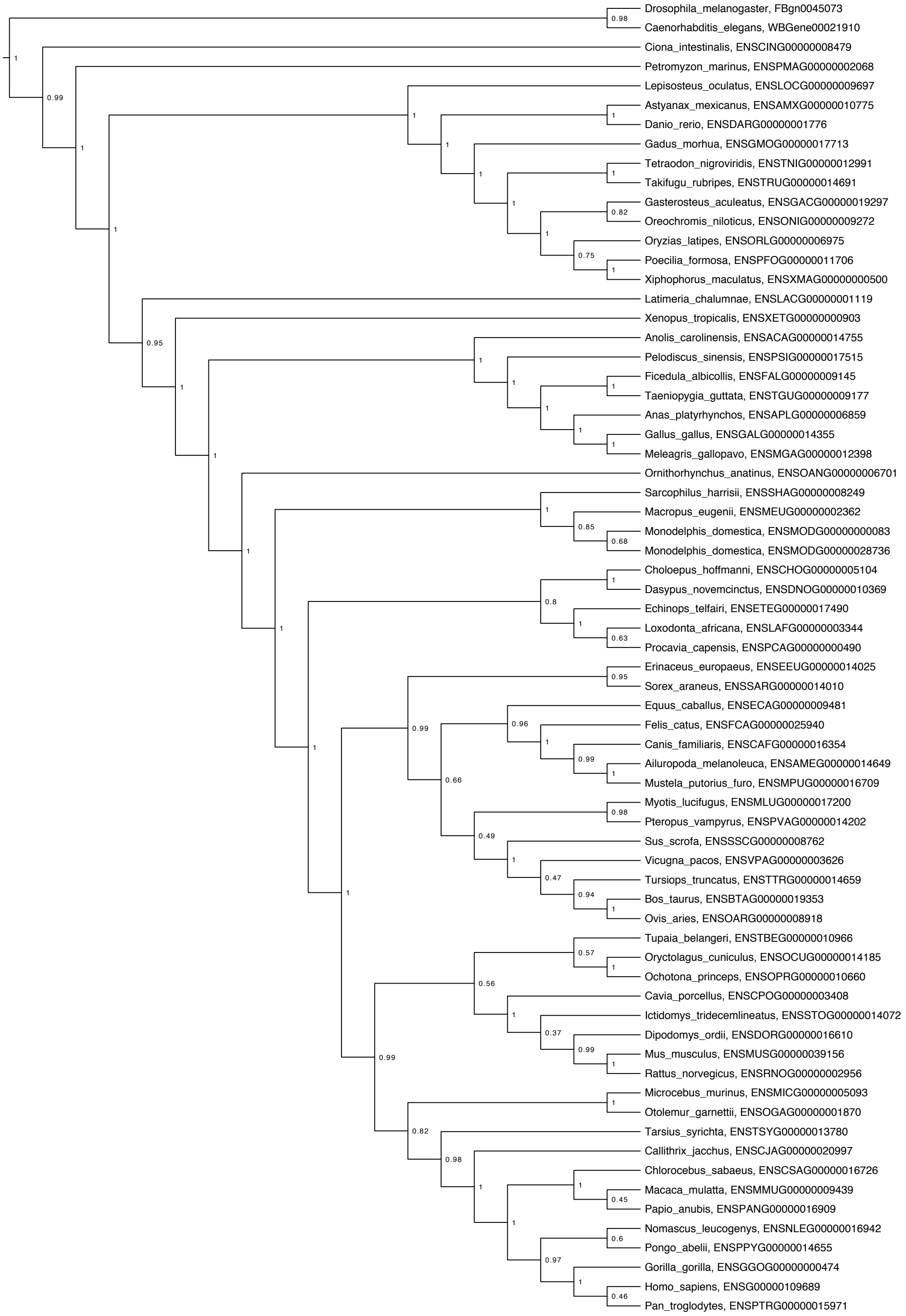

Supplement: Supplementary file 2 — STIM gene family and orthologous gene trees. (PDF 403 kb) [file 12859_2017_1850_MOESM2_ESM.pdf]
