# Supplementary material for: Integrated pipeline for inferring the evolutionary history of a gene family embedded in the species tree: a case study on the STIMATE gene family
Source: BMC Bioinformatics. 2017 Oct 3;18:439. doi: 10.1186/s12859-017-1850-2 (PMC5627428; doi:10.1186/s12859-017-1850-2)
Supplement: Supplementary file 4 — Dated species tree of 69 species. (PDF 61 kb) [file 12859_2017_1850_MOESM4_ESM.pdf]

Dated species tree of 69 species

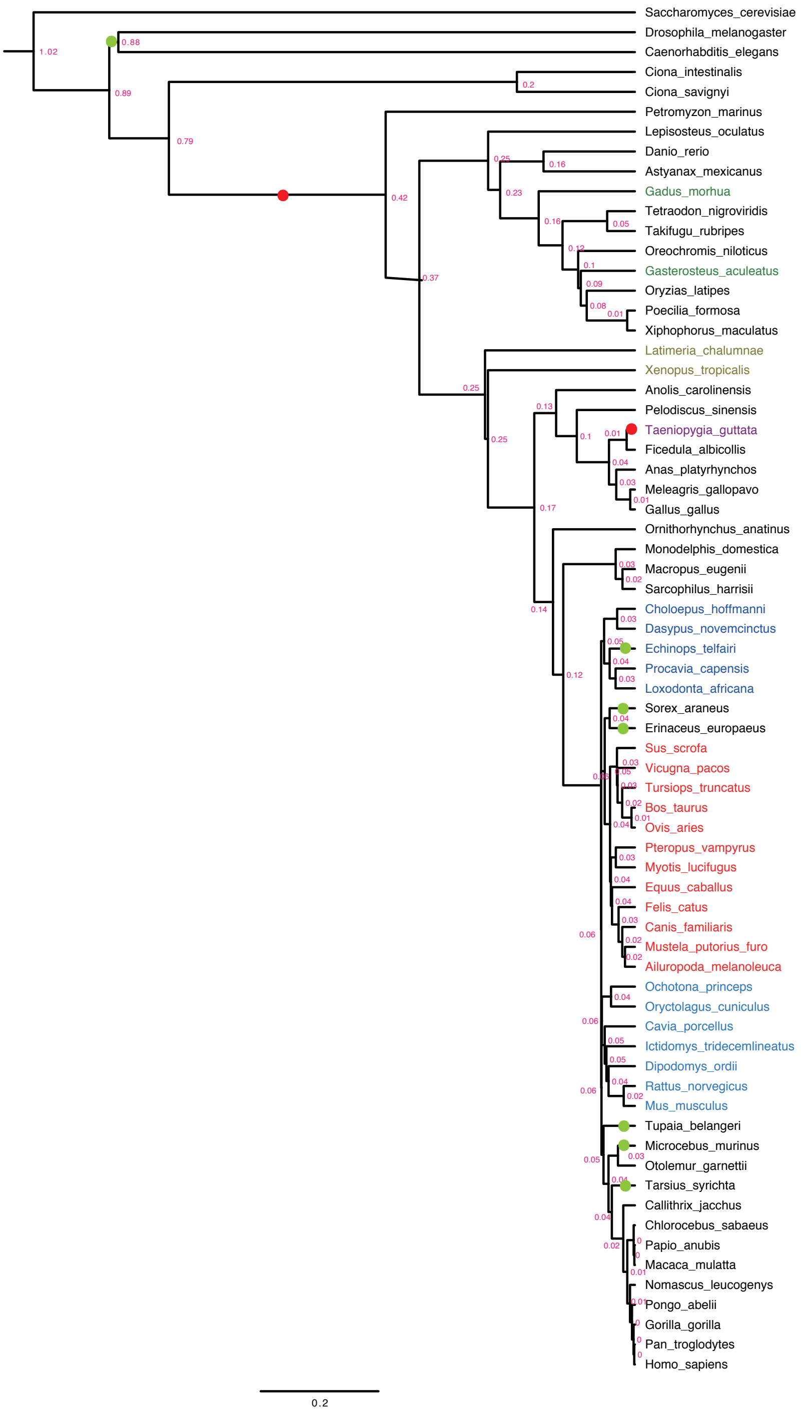

The node labels are the posterior probabilities with two digits remained after the decimal point, which was generated in *BEAST. We also highlighted (in colors other than black) the names of species displaying incongerence with STIMATE gene family trees on this species tree. The red and green dots on the branches are indicating the STIMATE-like gene duplications and losses, respectively.
