# Supplementary material for: Integrated pipeline for inferring the evolutionary history of a gene family embedded in the species tree: a case study on the STIMATE gene family
Source: BMC Bioinformatics. 2017 Oct 3;18:439. doi: 10.1186/s12859-017-1850-2 (PMC5627428; doi:10.1186/s12859-017-1850-2)
Supplement: Supplementary file 5 — Evolutionary history of the STIMATE gene family. (PDF 4081 kb) [file 12859_2017_1850_MOESM5_ESM.pdf]

Evolutionary history of the STIMATE gene family

As mentioned in Figure 3， based the phylogenetic trees inferred by our pipeline, we found a gene duplication event occurred at the beginning of STIMATE gene family evolutionary history on vertebrate genomes. It resulted in two copies of STIMATE-like genes on most fish and birds genomes. The STIMATEL gene was then lost in genomes of mammals and lampreys.

Then, as sequence alignment in Figure S1, some STIMATE-like genes from genomes of mammals have a tail aligned to the MUSTN1 sequence on the human STIMATE-MUSTN1 gene. These STIMATE-like genes are more likely to be STIMATE-MUSTN1 read-through. We cut-off these putative MUSTN1 sequence columns from our MSA before phylogenetic tree inferences to avoid the effect of MUSTN1 sequences. And this finding indicated that STIMATE might have a functional relationship with MUSTN1 in mammals.

After phylogenetic inference, by comparing the STIMATE gene family trees (Tree 2, Tree 3 in Figure 2) with the species tree, we identified several clades showing phylogenetic incongruence. Previous study indicated that there are various biological factors (lineage sorting, horizontal gene transfer, gene duplication and loss, hybridization, recombination natural selection and other more complex mechanisms) can cause phylogenetic incongruence[1]. Combined with the gene family trees of STIMATE's two co-players (STIM and ORAI)(additional file 2, additional file3)，we discuss the evolutionary history of STIMATE here.

The clades colored in maraschino on the three gene trees (Tree 1, 2, 3) are reflecting evolutionary relationship of STIMATE genes from 12 *Laurasiatheria*. The STIMATE genes on *Canis familiaris, Equus caballus, Sus scrofa* and *Vicugna pacos* show a most recent common ancestor, which is different from the species evolutionary history. Except for this, STIMATE genes from the other eight *Laurasiatheria* show a consistent relationship between Tree 1 and Tree 3 but incongruent from species tree and Tree 2. We thought ILS might be a better explain of this incongruence than the duplication and loss process. Because, the gene tree inferred under the multispecies coalescent model hold a consistent relationship reflected by sequence (Tree 1). In other words, the relationship of these 12 genes inferred under the ILS assumption got more supports from existing sequences than

that inferred under the duplication-loss assumption. In addition, the conserved STIMATE genomic neighborhoods across these species (Figure S2 in this file) retrieved from Genomicus v84[2, 3] also favors the ILS assumption.

Then, the clade colored in clover on Tree 1, Tree 2 and Tree 3 also show incongruence with the species tree. This incongruence occurred on the STIMATE's part on all these three trees. However, it is only occurred on the STIMATEL's part on Tree 1. Meanwhile, this incongruence was observed between its co-players' (STIM and ORAI) gene trees and species tree. As it occurred on phylogenetic trees of paralogs and putative co-players, we thought this incongruence might be a result from selection. For this incongruence only occurred on the STIMATEL' part on Tree 1(based on sequence only)， STIMATEL might undergo a weaker selection than STIMATE.

STIMATE genes from *Rodentia* genomes (colored in aqua) are located on totally different places on the three gene trees. They show a closer relationship with the STIMATE genes from genomes of mammals in *Xenarthra* and *Afrotheria* (colored in blueberry). However, *Rodentai* and *Atlantogenata* (including *Xenarthra* and *Afrotheria*) show a far distance on the species tree. Such incongruence also happened on other gene family trees [4]. We thought more complex evolutionary processes that we haven't taken into account, such as hybridization, might cause it.

As a special case, there are four STIMATE-like genes (colored in plum) on the genome of *Taeniopygia guttata.* Three of them are locating on the STIMATE orthologous gene tree. According to the CDS MSA (Figure S1 in this file), these three STIMATE genes (IDs: ENSTGUG00000013661, ENSTGUG00000005534 and ENSTGUG00000005534) show shorter CDS lengths than others. Two (IDs: ENSTGUG00000013661 and ENSTGUG00000005534) of them have most recent common ancestor, which indicating a species-specific duplication. Then, their genomic locations show that ENSTGUG00000005534 and ENSTGUG00000005538 are neighboring genes. And the sequence of ENSTGUG00000005534 and ENSTGUG00000005538 happened to constitute a full length STIMATE. So we thought ENSTGUG00000005534 and ENSTGUG00000005538 might be one gene with wrong annotation. From the Ensembl, we found it annotated 'split genes' to ENSTGUG00000005534 and ENSTGUG00000005538, which means these two genes actually one gene. Above all, as a result of species-specific gene duplication,

there are actually two STIMATE genes and one STIMATEL gene on the genome of *Taeniopygia guttata*.

Xenopus_tropicalis and *Latimeria_chalumnae* (colored in asparagus) show incongruent phylogenetic positions between the STIMATE gene family trees and the species tree. As we obtained consistent incongruence from the three gene family trees of STIMATE, it is hard to distinguish its causes.

Above all, based on the gene family trees inferred under our pipeline, we got some useful information of the STIMATE gene family evolutionary history. And we will integrate other data to verify these putative evolutionary events in the future.

Figure S1. The protein MSA of STIMATE

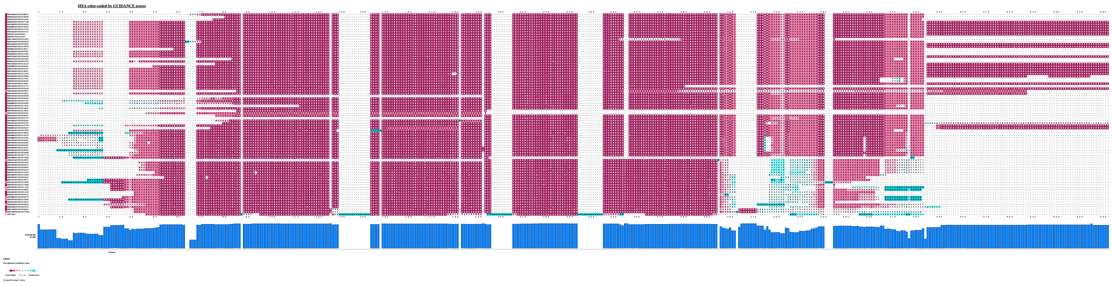

Figure S2. Conserved STIMATE genomic neighborhoods across different species. The STIMATE (TMEM110) is positioned in the centre of the display, intersecting with the solid line. The species tree is shown on the left of the aligned genomes, and by 18 genes on either side of STIMATE (TMEM110) is shown in the reference species, with different colors. Orthologs of these genes in other species are shown in matching colors.

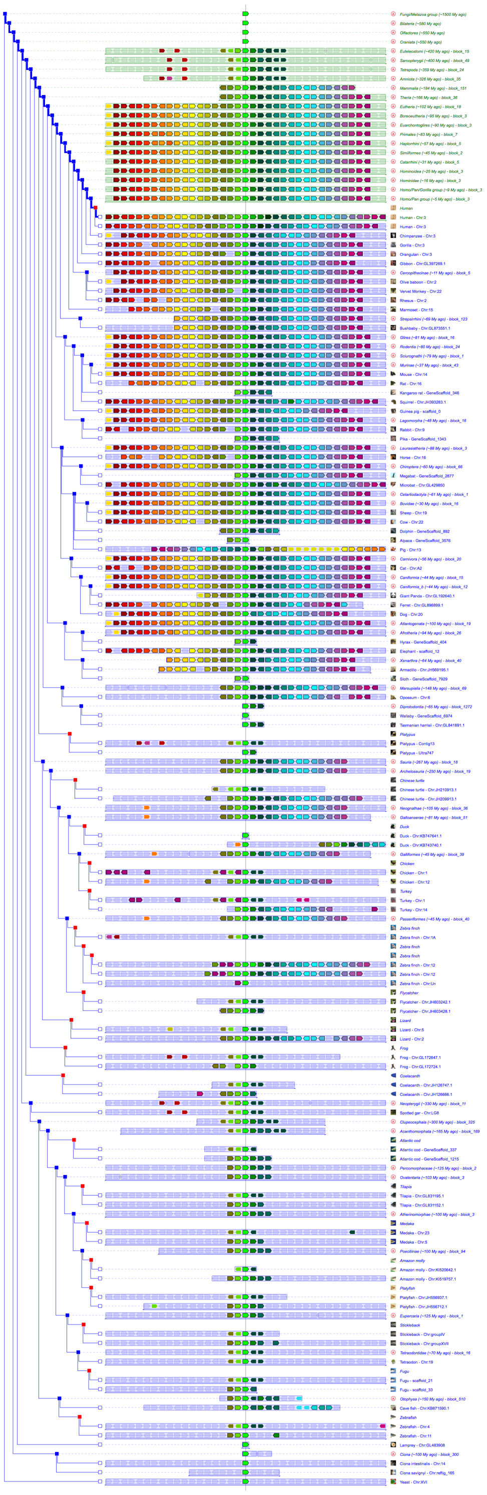

References

1. Som A: **Causes, consequences and solutions of phylogenetic incongruence**. *Briefings in Bioinformatics* 2015, **16**(3):536-548.
2. Louis A, Muffato M, Crollius HR: **Genomicus: five genome browsers for comparative genomics in eukaryota**. *Nucleic acids research* 2013, **41**(D1):D700-D705.
3. Louis A, Nga Thi Thuy N, Muffato M, Crollius HR: **Genomicus update 2015: KaryoView and MatrixView provide a genome-wide perspective to multispecies comparative genomics**. *Nucleic acids research* 2015, **43**(D1):D682-D689.
4. Wu XM, Wu FH, Wang XQ, Wang LL, Siedow JN, Zhang WG, Pei ZM: **Molecular evolutionary and structural analysis of the cytosolic DNA sensor cGAS and STING**. *Nucleic acids research* 2014, **42**(13):8243-8257.
