# Supplementary material for: Integrated pipeline for inferring the evolutionary history of a gene family embedded in the species tree: a case study on the STIMATE gene family
Source: BMC Bioinformatics. 2017 Oct 3;18:439. doi: 10.1186/s12859-017-1850-2 (PMC5627428; doi:10.1186/s12859-017-1850-2)
Supplement: Supplementary file 6 — Alignment filtering cutoff choice and comparison. (PDF 2385 kb) [file 12859_2017_1850_MOESM6_ESM.pdf]

Does sequence alignment require filtering?

In order to investigate whether we need to filter MSA in our pipeline, basing on the guidance score we selected several different cutoff values to lead the sequence filtering. First, we removed the putative unreliable columns using different guidance score cutoff values. Based on these filtered MSAs (including MSA without filtering), genetic tree sample sets (size: 20,000) from the posterior distribution were generated by using BEAST. Then the maximum clade credibility tree was summarized in TreeAnnotator with 10% burn-in samples for each tree sample set. Finally, we compared these trees according to their topology log likelihood based on the original MSA and the RF distances from species tree (Table S1).

When we set the cutoff with small values like 0.3 and 0.5 to filter columns with very low robustness, we had a consistent gene tree with that inferred from MSA without filtering. When the cutoff was set to a larger value like 0.8 or 0.93 (default for guidance 2), the resulted trees are different from the tree based on no filtering MSA. As Table 1, we found the trees represent high cutoff values show lower log likelihood calculated based on the original MSA. Meanwhile, these trees show larger average normalized RF distances from species tree.

Therefore, as previous study**[1]** shows that improper filtering of MSA may lead to wrong gene trees. We thought that setting appropriate cutoff values for filtering sequence alignment might be desirable and thus reduce our computation time. Here, our pipeline integrated GUIDANCE 2 as an alignment tool, which give visualization of the alignment. We can quickly choose a suitable cutoff value according to the visualization. And we display an example of such alignment graph in Figure S3 below. In addition, our code provides a Perl program that allows us to set the right cutoff values for rapid filtering based on alignment and scores in GUIDANCE 2's results. If there is no need to filter, we can get the original MSA without filtering by skipping this step.

Table S1. Gene tree likelihood based on MSA (without filtering) and RF distance from species tree

Cutoff values	No filtering	0.3	0.5	0.8	0.93
**RF**	0.31	0.31	0.31	0.34	0.38
**Log likelihood**	-28960	-28960	-28960	-29017	-29129

Figure S3. An example of the visualization of alignment

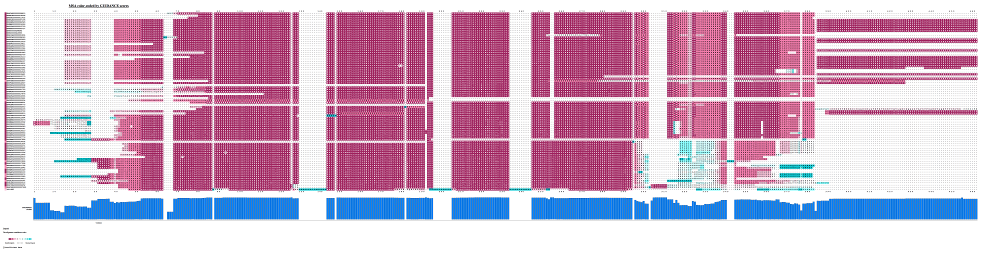

1. Tan G, Muffato M, Ledergerber C, Herrero J, Goldman N, Gil M, Dessimoz C: **Current Methods for Automated Filtering of Multiple Sequence Alignments Frequently Worsen Single-Gene Phylogenetic Inference**. *Systematic biology* 2015, **64**(5):778-791.
