# Supplementary material for: Integrated pipeline for inferring the evolutionary history of a gene family embedded in the species tree: a case study on the STIMATE gene family
Source: BMC Bioinformatics. 2017 Oct 3;18:439. doi: 10.1186/s12859-017-1850-2 (PMC5627428; doi:10.1186/s12859-017-1850-2)
Supplement: Supplementary file 7 — Comparison with Phylobayes and TERA. (PDF 51 kb) [file 12859_2017_1850_MOESM7_ESM.pdf]

Comparison with other phylogenetic algorithms

We replaced BEAST with Phylobayes to get a gene tree sample set with 20,000 trees. In our test, Phylobayes needs more time to produce such a set. Then we summarized the maximum clade credibility tree for the gene tree set with 25% burn-in samples discarded resulted from Phylobayes and BEAST respectively. We found there is about 0.22 normalized RF (Robinson-Foulds) distance between the resulted two trees. Based on these tree sets generated by BEAST and Phylobayes, ALE output two gene family trees with 0.13 normalized RF distance. We found that, although the different tools used in the first step may result in different gene tree sets, they affect a little on the ALE results in the second step. In addition, based on the two inferred trees from ALE, there is a consistent orthologous relationship between them.

TERA is a parsimony-based algorithm using a similar idea as ALE. We replaced ALE with TERA to get the gene family tree. It derived a gene family tree with 0.14 RF distance from the result of ALE. However, their orthologous relationships are consistent. Compared with the species tree, the TERA tree has about 0.17 average normalized RF distances, which is greater than the distance between the species tree and the ALE tree (~ 0.12). Meanwhile, based on the CDS MSA generated by our pipeline, ALE output a gene family tree with higher maximum log likelihood (log likelihood: -28479.388) than TERA (log likelihood: -28479.388).
